# The Association of VDAC with Cell Viability of PC12 Model of Huntington’s Disease

**DOI:** 10.3389/fonc.2016.00238

**Published:** 2016-11-11

**Authors:** Andonis Karachitos, Daria Grobys, Klaudia Kulczyńska, Adrian Sobusiak, Hanna Kmita

**Affiliations:** ^1^Laboratory of Bioenergetics, Faculty of Biology, Institute of Molecular Biology and Biotechnology, Adam Mickiewicz University in Poznań, Poznań, Poland

**Keywords:** VDAC protein, Huntington’s disease, mitochondria, huntingtin, intact cells, status of mitochondrial coupling

## Abstract

It is becoming increasingly apparent that mitochondria dysfunction plays an important role in the pathogenesis of Huntington’s disease (HD), but the underlying mechanism is still elusive. Thus, there is a still need for further studies concerning the upstream events in the mitochondria dysfunction that could contribute to cell death observed in HD. Taking into account the fundamental role of the voltage-dependent anion-selective channel (VDAC) in mitochondria functioning, it is reasonable to consider the channel as a crucial element in HD etiology. Therefore, we applied inducible PC12 cell model of HD to determine the relationship between the effect of expression of wild type and mutant huntingtin (Htt and mHtt, respectively) on cell survival and mitochondria functioning in intact cells under conditions of undergoing cell divisions. Because after 48 h of Htt and mHtt expression differences in mitochondria functioning co-occurred with differences in the cell viability, we decided to estimate the effect of Htt and mHtt expression lasted for 48 h on VDAC functioning. Therefore, we isolated VDAC from the cells and tested the preparations by black lipid membrane system. We observed that the expression of mHtt, but not Htt, resulted in changes of the open state conductance and voltage-dependence when compared to control cells cultured in the absence of the expression. Importantly, for all the VDAC preparations, we observed a dominant quantitative content of VDAC1, and the quantitative relationships between VDAC isoforms were not changed by Htt and mHtt expression. Thus, Htt and mHtt-mediated functional changes of VDAC, being predominantly VDAC1, which occur shortly after these protein appearances in cells, may result in differences concerning mitochondria functioning and viability of cells expressing Htt and mHtt. The assumption is important for better understanding of cytotoxicity as well as cytoprotection mechanisms of potential clinical application.

## Introduction

Huntington’s disease (HD) is a fatal neurodegenerative disease, which is characterized by progressive cognitive deterioration, psychiatric disturbances, and movement disorder with maximum degeneration occurring in striatum and deep layers of the cerebral cortex [e.g., Bonelli and Hofmann (1); Gövert and Schneider (2); Glajch and Sadri-Vakili (3)]. The disease is caused by the expansion of the unstable trinucleotide CAG (glutamine codon) repeat region within the first exon of the gene encoding the protein huntingtin (Htt) that results in synthesis of its mutant form (mHtt) containing a sequence of 36 or more glutamines at N-terminus (4). The mechanism by which mHtt causes HD is still unknown although available data suggest that mitochondrial defects initiate the disease onset (5–10). Accordingly, over 130 review papers on the role of mitochondria in HD pathogenesis have been published till now. They address mHtt effects on mitochondrial bioenergetics and biogenesis, protein import complex assembly, fission and fusion, mitochondrial transport including Ca^2+^ and metal homeostasis, and the degradation of damaged mitochondria *via* autophagy (mitophagy). Simultaneously, it is also evident that voltage-dependent anion-selective channel (VDAC), regarded as a dynamic regulator, or even governor, of mitochondrial functions, contributes to affected phenomena directly or by interacting with the involved proteins [e.g., Colombini (11); Mannella and Kinnally (12); Shoshan-Barmatz et al. (13); Maldonado and Lemasters (14); Martel et al. (15); Karachitos et al. (16)].

Because HD inheritance is autosomal dominant, the prevailing view is that mHtt-mediated symptoms result from a toxic gain-of-function mechanism although loss-of-function mechanisms for both mHtt and Htt are also proposed (17, 18). Htt is localized mainly in cytoplasm and is known to exhibit anti-apoptotic properties (19) and to be required for mammalian embryogenesis and neurogenesis (20, 21). The domain model of Htt indicates the presence of domains important for protein interactions. Indeed, numerous reports indicate that Htt interacts with about 200 proteins, which represent a diverse array of biological functions, including synaptic transmission, cytoskeletal organization, signal transduction, gene expression regulation, and metabolism. Importantly, the interaction was also reported for human VDAC isoforms [e.g., Kaltenbach et al. (22); see also http://thebiogrid.org]. Thus, one could assume that the possible upstream events in mitochondria dysfunction resulting in HD may include VDAC (16).

Resolving the role of VDAC in HD pathogenesis may be important for the development of new therapeutic strategies concerning the disease as well as other neurodegenerative diseases. Moreover, the obtained results may also contribute to better understanding of VDAC role in cell death and to the development of efficient anti-cancer strategies. Accordingly, available data support an important role of VDAC in cancer cell survival [e.g., Maldonado and Lemasters (14); Leanza et al. (23); Shoshan-Barmatz et al. (24)]. Here, we used inducible PC12 cell model of HD, i.e., PC12 HD-Q23 and PC12 HD-Q74 cycling cells, to determine the effect of short-term expression of Htt and mHtt on the cell viability and mitochondria functioning in intact cells as well as properties of VDAC isolated from the cells differing in viability and mitochondrial coupling status. The obtained results indicate that when compared with Htt, the cytotoxic effect of mHtt on PC12 cells is preceded by its different impact on the cell respiration and coincides with its different influence on the cell status of mitochondrial coupling and reconstituted VDAC properties.

## Materials and Methods

### Cell Lines and Growth Conditions

PC12 cell lines were derived from pheochromocytoma of the rat adrenal medulla (25). PC12 HD-Q23 and PC12 HD-Q74 cells expressing exon 1 of the gene encoding huntingtin with 23 (Htt) or 74 (mHtt) repeats of glutamine residues were kindly provided by David Rubinsztein and Andreas Wyttenbach (University of Cambridge, UK). The cells were grown in TPP tissue culture T75 flasks, in the recommended high glucose (4.5 g/l) DMEM (Dulbecco’s Modified Eagle’s Medium) supplemented with 10% heat-inactivated horse serum, 5% fetal bovine serum, 2 mM l-glutamine, 100 units/ml penicillin G, 100 μg/ml streptomycin sulfate, and 0.25 μg/ml amphotericin B, in a humidified 5% CO_2_ atmosphere at 37°C (Thermo Scientific Heracell 150i CO_2_ incubator). Poly-l-ornithine hydrobromide (10 μg/ml) was applied as a coating reagent for culture flasks and plates. Expression of Htt and mHtt was induced by 1 μg/ml doxycycline and monitored by their labeling with GFP (Figure S1 in Supplementary Material) fused to N-terminus of the exon 1. The expression was performed in DMEM without antibiotic and antimycotic compounds, for different period of time (from 4 to 48 h), and at cell confluence of approximately 80–90%.

### Estimation of Cell Viability

Viability of PC12 HD-Q23 and PC12 HD-Q74 cells was estimated in 24-well plates used for the cell culturing for a given period of time in the presence or absence of Htt and mHtt expression, respectively. The number of PC12 cells adhering to the bottom of the plates was counted using Zeiss Axiovert40 phase-contrast microscope. To quantify results of the MTT cell proliferation colorimetric assay, UV-1650PC Shimadzu spectrophotometer (λ = 570 nm) was applied. The assay is based on 3-[4,5-dimethylthiazol-2-yl]-2,5-diphenyltetrazolium bromide (thiazolyl blue – MTT) conversion to an insoluble purple formazan by cleavage of the tetrazolium ring by living cell dehydrogenase enzymes (26). After the proper duration of Htt and mHtt expression and control cultures without the expression, cells were incubated with MTT at a concentration of 0.25 mg per 1 ml of DMEM for 3 h at 37°C, and the created formazan was dissolved in acidic isopropanol (0.04M HCl in absolute isopropanol). To estimate the effect of Htt or mHtt, the change index was calculated between cells expressing Htt or mHtt and control cells, i.e., cultured for the same time but without Htt or mHtt expression.

### Measurements of Cell Respiration and Status of Mitochondrial Coupling in Intact Cells

The cells expressing Htt or mHtt and control cells were washed and suspended in phosphate-buffered saline (PBS), pH 7.4. The rate of oxygen uptake by cells (2 × 10^6^) was measured at 37°C in 0.4 ml of high glucose DMEM without antibiotic and antimycotic compounds, in a water-thermostated incubation chamber with a computer-controlled Clark-type O_2_ electrode (Oxygraph, Hansatech, UK) (27–30). State 4 (oligomycin-resistant respiration) was enforced by addition of 2 μg/ml oligomycin (an inhibitor of mitochondrial ATPase), whereas state U (uncoupled state) was induced by FCCP (carbonyl cyanide p-trifluoromethoxyphenylhydrazone; an uncoupler) added in portions of 0.1 μM each to avoid respiration inhibition (Figure 2A). To estimate the status of mitochondrial coupling in intact cells, basal respiration, contribution of state 3 to basal respiration, and uncoupling capacity of FCCP were calculated (29, 31). The state 3 (oligomycin-inhabitable respiration) was calculated by subtracting state 4 from basal respiration. The contribution of state 3 to basal respiration was calculated as the ratio of state 3 to basal respiration and corresponds to mitochondrial coupling efficiency. The uncoupling capacity of FCCP denotes the state U to state 4 ratio and corresponds to mitochondrial coupling capacity. To evaluate the effect of Htt or mHtt, the change index for the parameters was calculated between cells expressing Htt or mHtt and control cells (cultured for the same time but without Htt or mHtt expression).

### Isolation of VDAC Proteins from PC12 Cells

Isolation of mitochondria was based on Gross et al. (32). After washing with PBS, PC12 cell pellet was transferred to Falcon tubes and washed twice with PBS at 4°C (10 min, 500 × *g*). The pellet was suspended in medium A (20 ml per 1 g of cells) containing 10 mM saccharose, 200 mM mannitol, 5 mM Hepes-KOH (pH 7.4), 1 mM EGTA, 1 mM EDTA and protease inhibitor mix (Lab Empire, PIC002.1), homogenized with Dounce homogenizer, and centrifuged at 4°C, 10 min, 900 × *g*. The obtained supernatant was centrifuged at 4°C, 10 min, 13,000 × *g*, and the consecutive pellet was suspended in medium B containing 10 mM sacharose, 200 mM mannitol, 5 mM Hepes-KOH (pH 7.4), and 1 mM EGTA. The suspension was centrifuged at 4°C, 10 min, 900 × *g*, and the obtained supernatant was centrifuged at 4°C, 10 min, 13,000 × *g* and then washed once in medium B. To isolate VDAC proteins, the pellet of isolated mitochondria was suspended in the solubilization buffer containing 3% Triton X-100, 10 mM Tris–HCl (pH 7.0), and 1 mM EGTA and was incubated for 30 min on ice. Then, 0.6 ml of the suspension was loaded onto a dry hydroxyapatite/celite column (33), and three fractions of 200 μl were collected. The presence of human VDAC isoforms were confirmed by liquid chromatography coupled to tandem mass spectrometry (LC–MS/MS) performed in the Laboratory of Mass Spectrometry, Institute of Biochemistry and Biophysics, Polish Academy of Sciences (Warsaw, Poland). The LC–MS/MS data, including the Exponentially Modified Protein Abundance Index (emPAI), were also used to indicate quantitative relations between the isoforms and to detect proteins present in the obtained VDAC preparations.

### Conductance Measurements in Black Lipid Membrane System

Artificial phospholipid membranes were formed from soybean asolectin (Avanti Polar Lipids, purified by acetone precipitation before use) suspended at concentration of 25 mg/ml in n-decane, across a circular hole (aperture diameter about 250 μm) in the thin wall of Delrin chamber (Warner Instruments) separating two compartments (cis–trans) filled with unbuffered 1M KCl (31, 34, 35). The pH of KCl solution was 6.9. The chamber was connected to the recording equipment through a matched pair of Ag–AgCl electrodes. About 0.1–0.5 μg of VDAC preparations were added to the *cis* compartment after membrane formation. *Cis* also refers to the compartment where the voltage was held. The voltage-dependence was determined due to current measurements probed with triangular voltage waves (10 mHz in frequency and 60 mV in amplitude). Signals were amplified by BLM-120 bilayer amplifier (Bio-LOGIC Science Instruments), and computer software was used for data collection. The average amount of analyzed insertion events for a given potential value and VDAC preparation was 45.

### Other Methods

Protein concentration was measured by the method of Bradford and albumin bovine serum (BSA), essentially fatty acid free, was used as a standard. Taking into account the increased absorption of the dye by BSA, in the case of isolated VDAC preparations the correction factor obtained due to application of BSA and ovalbumin as standards was applied. Expression of Htt and mHtt was monitored by their labeling with GFP and ZEISS OBSERVER.Z1 fluorescence microscope.

## Results

### Viability of PC12 Cells Expressing Htt or mHtt and Their Status of Mitochondrial Coupling

To study HD pathogenesis, including the role of mitochondria, only the first exon of wild type and mutant huntingtin (Htt and mHtt, respectively) encoding genes are often applied because N-terminus fragments of the mutant protein have been shown to recapitulate several aspects of the full length protein toxicity (36). However, the role of mitochondria dysfunction is randomly studied in intact cells although the available data highlight the importance of the cellular environment (30).

**Figure 1:**
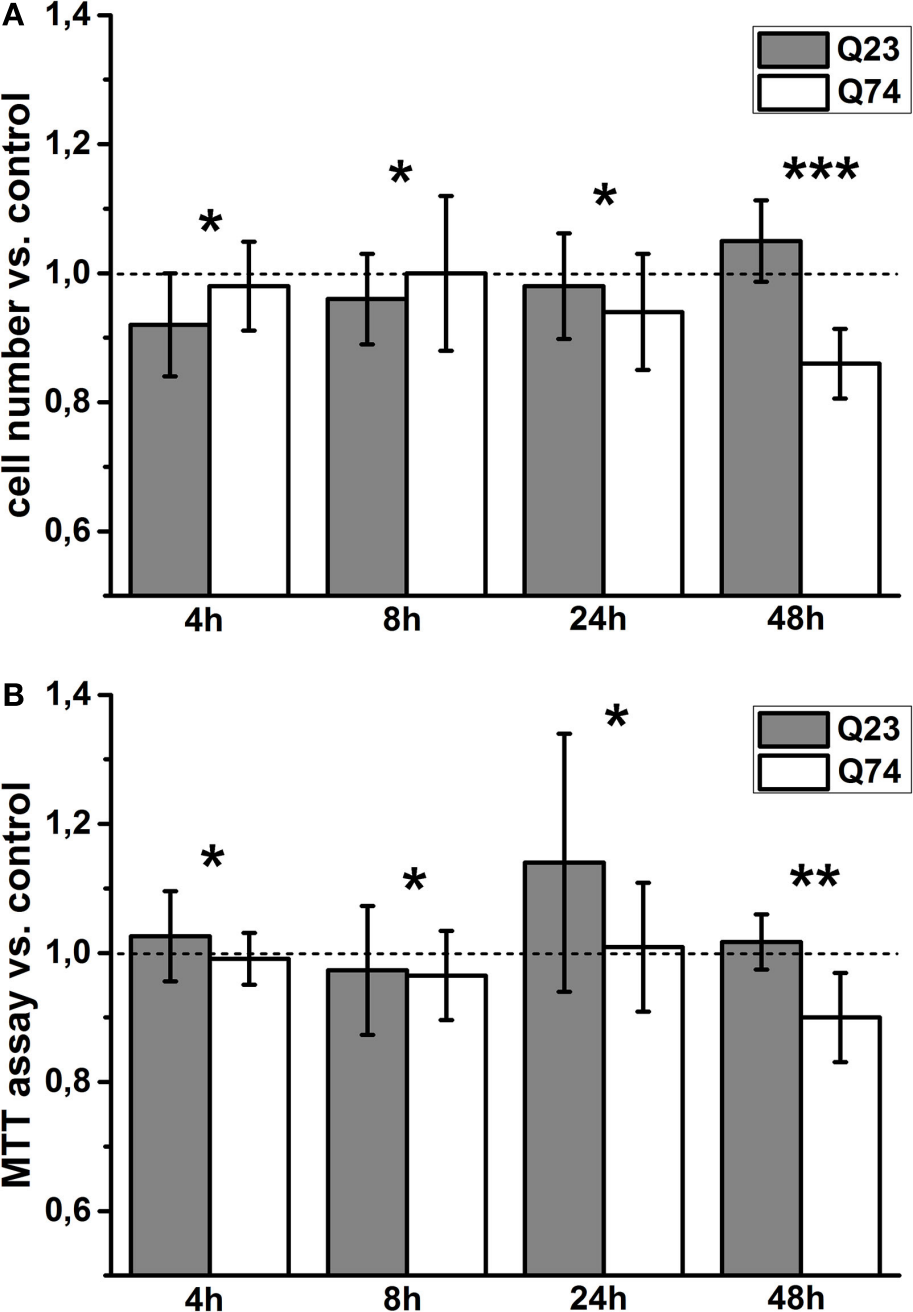
**Viability changes of PC12 HD-Q23 and PC12 HD-Q74 cells with expression of Htt and mHtt, respectively, induced for a given period of time**. Cells were cultured in 24-well plates, and the expression of Htt and mHtt was performed for a given period of time. The change index for **(A)** cell number adhering to the bottom of the plates and **(B)** the MTT cell proliferation colorimetric assay. The change index was calculated between cells expressing Htt or mHtt and control cells, i.e., cultured for the same time but without Htt or mHtt expression. The data are presented as mean values ± SD of three independent experiments. Statistical analysis was based on *t*-test: * – not statistically significant, ** – not quite statistically significant (*p* = 0.067), *** – statistically significant (*p* < 0.05).

To monitor viability of PC12 cells expressing Htt and mHtt (PC12 HD-Q23 and PC12 HD-Q74, respectively) for the studied periods of time (4, 12, 24, and 48 h), the number of cells that adhere to the bottom of the applied plates was calculated [e.g., Rantzsch et al. (37)], and the data were verified by the application of MTT assay based on metabolic activity of living cells [e.g., Kupcsik (38)]. Then, the studied parameter values were used to calculate the change index between cells with and without expression of Htt and mHtt (Figure 1). Thus, the index value higher or smaller than 1 denotes an increase or a decrease in viability of cells with a given protein expression (i.e., Htt and mHtt) when compared with proper control cells (i.e., without induced expression of Htt and mHtt) cultured for the same period of time. The obtained results indicated that a clear difference in viability of PC12 HD-Q23 and PC12 HD-Q74 cells occurred when Htt and mHtt expression lasted for 48 h. Namely, the expression of Htt did not appear to affect PC12 HD-Q23 cell viability, but viability of PC12 HD-Q74 cells decreased.

**Figure 2:**
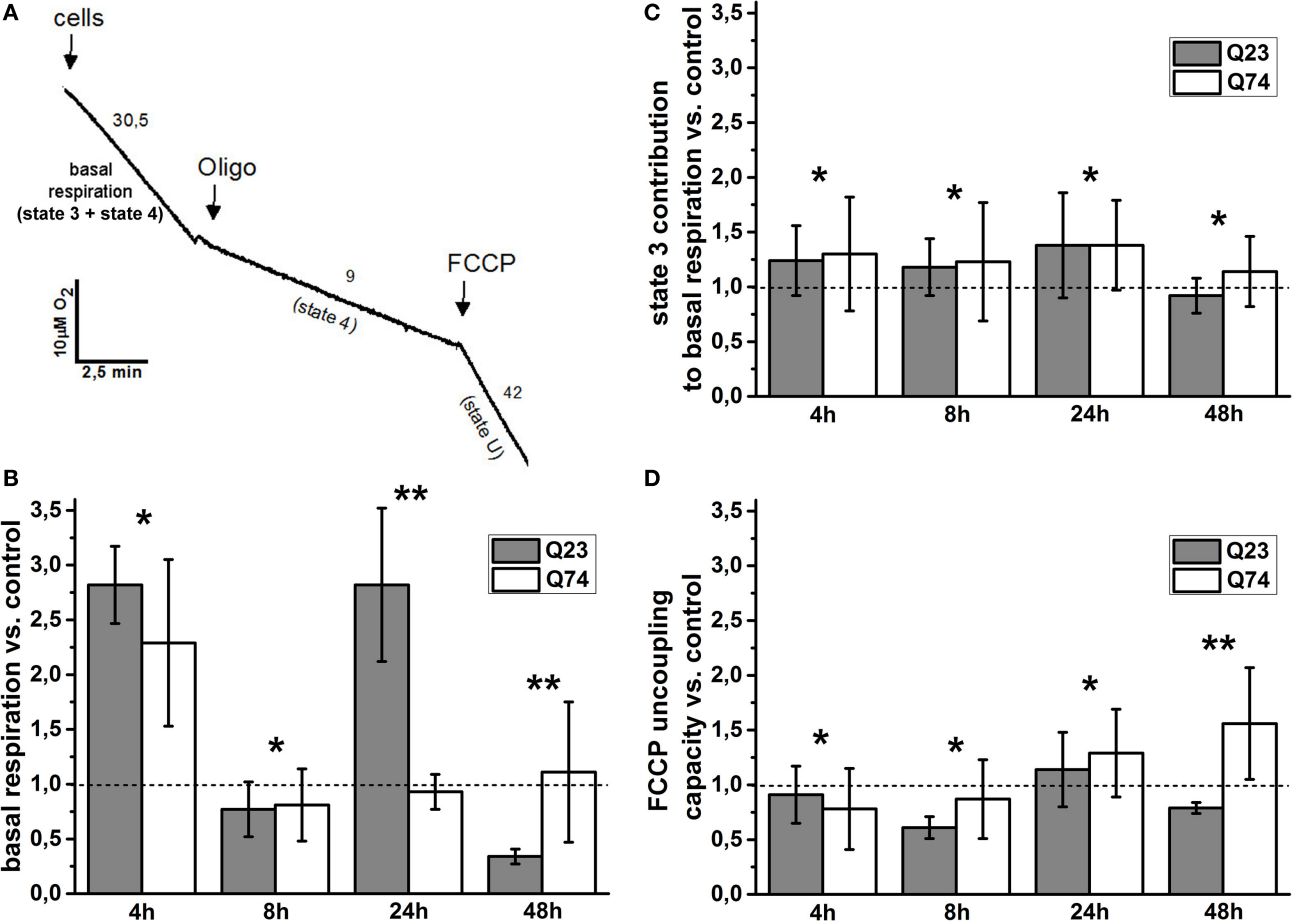
**Changes of the mitochondrial coupling status in intact PC12 HD-Q23 and PC12 HD-Q74 cells with expression of Htt and mHtt, respectively, induced for a given period of time**. **(A)** A representative trace applied for further calculations. The rate of oxygen uptake by cells (2 × 10^6^) was measured at 37°C in DMEM. Oligo, oligomycin. The calculated values of the change index for **(B)** basal respiration, **(C)** state 3 contribution to basal respiration, and **(D)** FCCP uncoupling capacity. The change index was calculated between cells expressing Htt or mHtt and control cells, i.e., cultured for the same time but without Htt or mHtt expression. State 4 was enforced by the addition of 2 μg/ml oligomycin, and state 3 was calculated by subtracting state 4 from basal respiration. The optimal concentration of FCCP yielding the highest respiratory rate (state U) was settled by the uncoupler addition in portions of 1 μM each. The FCCP uncoupling capacity denotes state U to state 4 ratio. The data are presented as mean values ± SD of three independent experiments. Statistical analysis was based on *t*-test: * – not statistically significant, ** – statistically significant (*p* < 0.05).

To estimate the role of mitochondria functional state for the observed difference, cell respiration and the status of mitochondrial coupling in intact PC12 HD-Q23 and PC12 HD-Q74 cells were determined. It is well known that the rate of cell respiration is controlled by the respiratory state of mitochondria, which fluctuates between the resting state (state 4; low oxygen uptake, the higher inner membrane potential) and the phosphorylating state (state 3; high oxygen uptake, the lower inner membrane potential). Thus, basal (actual) cell respiration oscillates between these two states (27, 28). The status of mitochondrial coupling in intact cells can be estimated by calculation of mitochondrial coupling efficiency and mitochondrial coupling capacity. As mentioned in Section “Materials and Methods,” they correspond to the state 3 contribution to basal respiration and FCCP uncoupling capacity (the state U to state 4 ratio), respectively (29, 31). State 3 can be calculated by subtracting state 4 from basal respiration, and state 4 can be enforced by inhibitors of adenine nucleotide translocase or ATP synthase able to cross the cell membrane (i.e., by elimination of state 3), whereas state U (uncoupled state that connotes a maximal rate of oxygen uptake) can be induced by FCCP, which collapses the inner membrane potential. A representative trace applied for the calculations is shown in Figure 2A, and the calculated original data are presented in Table S1 in Supplementary Material.

The basal respiration, state 3 contribution to basal respiration, and FCCP uncoupling capacity were calculated for PC12 HD-Q23 and PC12 HD-Q74 cells expressing Htt and mHtt for a given period of time (4, 12, 24, and 48 h), and then change index was determined for these parameters between the cells and their counterparts cultured for the same time but without expression of Htt and mHtt (control cells). Therefore, the change index value higher or smaller than 1 denotes an increase or a decrease in a value of the studied parameter for cells with expression of Htt or mHtt when compared with control cells, respectively.

The values of the change index for basal respiration were shown in Figure 2B. It was evident that the impact of Htt and mHtt expression on basal respiration including fluctuation effect was initially very similar when compared to the proper control cells. However, for the expression longer than 8 h, a clear difference occurred between PC12 HD-Q23 and PC12 HD-Q74 cells and concerned also the extent of the observed fluctuation. Namely, very weak, if any, changes of basal respiration were observed for PC12 HD-Q74 cells with mHtt expression lasted for 24 and 48 h when compared to control cells whereas for PC12 HD-Q23 cells with Htt expression lasted for 24 and 48 h, a strong increase and decrease of the basal respiration occurred when compared with control cells. To check whether the changes of basal respiration may result from changes of mitochondrial coupling efficiency, the contribution of state 3 to basal respiration was calculated and then compared between PC12 HD-Q23 and PC12 HD-Q74 cells with and without expression of Htt and mHtt, respectively, due to determination of the change index. As shown in Figure 2C, for the studied durations of Htt and mHtt expression, no important differences were observed for PC12 HD-Q23 and PC12 HD-Q74 cells when compared to the proper control cells, except for the expression lasted for 48 h when a slightly stronger decrease in the state 3 contribution to basal respiration was calculated for PC12 HD-Q23. Since actual mitochondrial coupling efficiency may result from the demand for ATP and/or mitochondrial coupling capacity, FCCP uncoupling capacity was calculated and compared for the studied cells to determine the change index (Figure 2D). Interestingly, no differences were observed for PC12 HD-Q23 and PC12 HD-Q74 cells when compared to the proper control cells except for Htt and mHtt expression performed for 48 h. In this case, a distinct decrease of FCCP uncoupling capacity was calculated for PC12 HD-Q23 cells whereas for PC12 HD-Q74 cells a slight increase of the parameter was obtained. Thus, the latter coincided with differences in mitochondrial coupling efficiency and the cell viability as determined by the proper change index values.

### VDAC Activity in PC12 Cells Expressing Htt or mHtt and Differing in the Status of Mitochondrial Coupling

**Figure 3:**
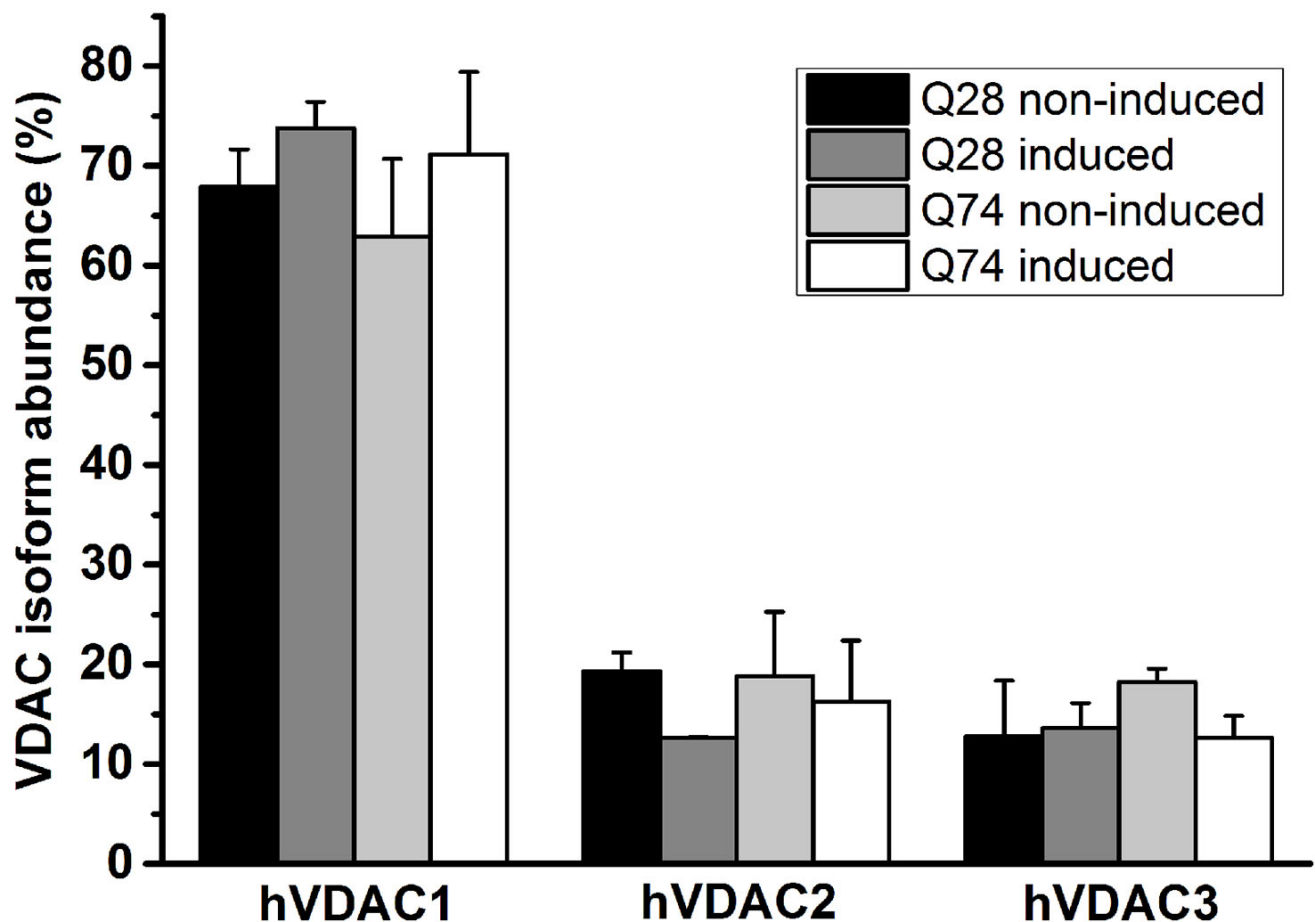
**Quantitation of VDAC isoforms in VDAC preparations isolated from PC12 HD-Q23 and PC12 HD-Q74 cells**. The quantitation was based on emPAI. The data are presented as mean values ± SD of three independent experiments. Q23 non-induced and Q74 non-induced denote PC12 HD-Q23 and PC12 HD-Q74 cells cultured for 48 h without expression of Htt and mHtt, respectively, whereas Q23 induced and Q74 induced denote the cells with the proper expression lasted for 48 h.

The differences in the status of mitochondrial coupling, observed for PC12 HD-Q23 and PC12 HD-Q74 cells with expression of Htt and mHtt lasted for 48 h, may result from differences in the permeability of the mitochondrial outer membrane. Therefore, VDAC was isolated from the cells cultured for 48 h under conditions of the presence and absence of Htt and mHtt expression to estimate the channel properties after reconstitution in artificial phospholipid membranes (BLM system). The analysis of the emPAI provided with LC–MS/MS data concerning the isolated VDAC preparations indicated that the most abundant VDAC isoform in PC12 HD-Q23 and PC12 HD-Q74 cells was VDAC1. The isoform represented about 65–75% of all VDAC proteins, whereas the contribution of VDAC2 and VDAC3 was smaller and comparable. Importantly, the quantitative relationships were not changed by Htt and mHtt expression performed for 48 h (Figure 3).

It is well known that VDAC behavior in planar phospholipid membranes is voltage-dependent and symmetrical [e.g., Benz (39); Colombini et al. (40)], meaning that the channel closes at about the same rate and to about the same extent depending on the applied potential value but regardless its sign. Thus, at lower voltages the reconstituted VDAC displays the ability to adopt a fully open state that is anion selective with a conductance of about 4 nS in 1M KCl and transits into multiple closed states of significantly smaller conductance and anionic selectivity and consequently changed permeability when the voltage is increased.

**Figure 4:**
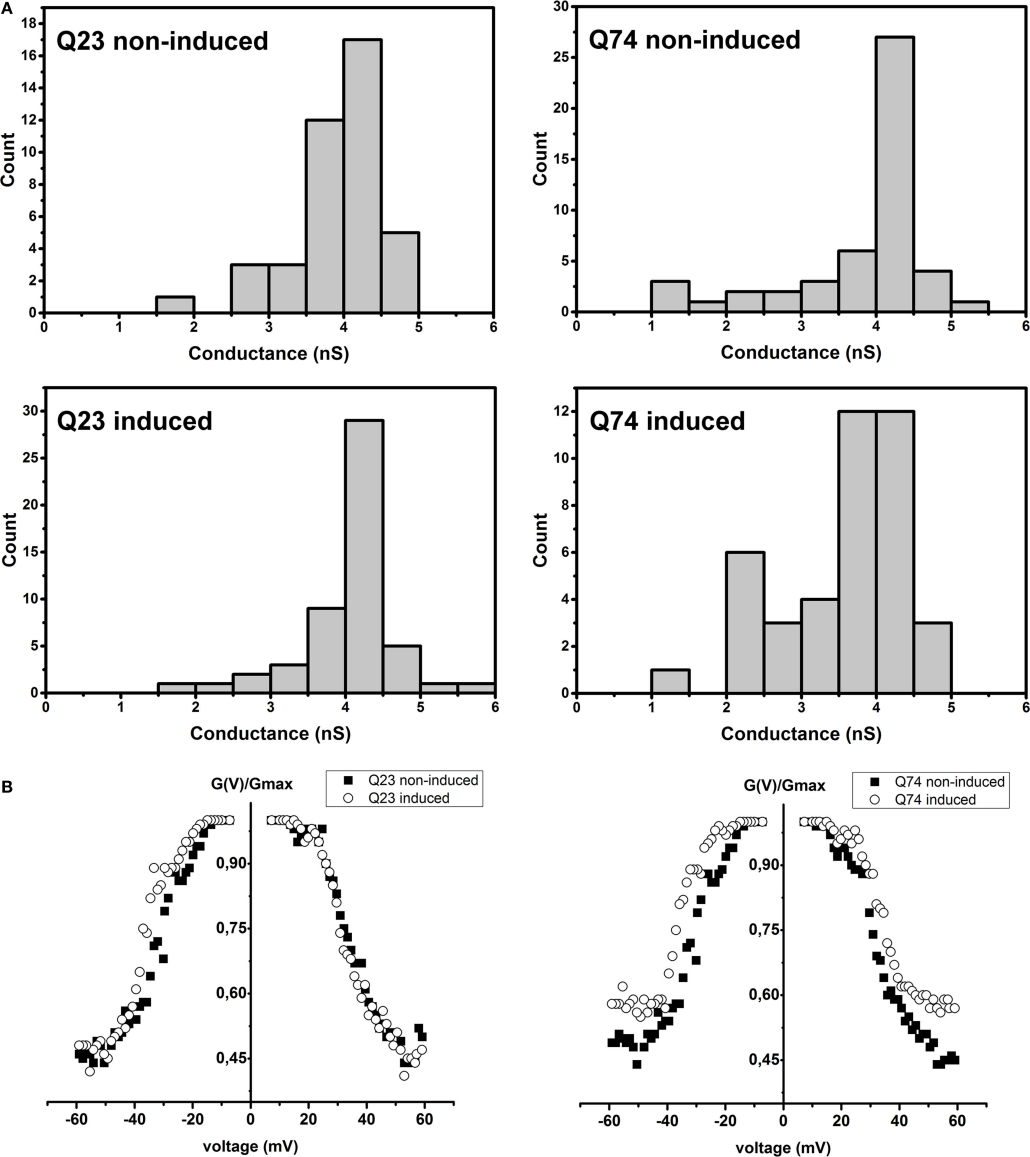
**The effect of Htt and mHtt expression on reconstituted VDAC preparations isolated from PC12 HD-Q23 and PC12 HD-Q74 cells, respectively, after the expression lasted for 48 h**. **(A)** Histograms of conductances calculated at a membrane potential of +10 mV. The data reflect the following number of insertion events: Q23 non-induced, 41; Q23 induced, 52; Q74 non-induced, 41, and Q74 induced, 49 **(B)** The voltage-dependence assessed due to current measurements by triangular voltage waves. *G*_max_ denotes average conductance values calculated in the presence of the lowest applied potential. The average amount of the analyzed insertions for a given potential value and VDAC preparation was 45 for 2–3 different preparations of VDAC proteins. Q23 non-induced and Q74 non-induced denote PC12 HD-Q23 and PC12 HD-Q74 cells cultured for 48 h without expression of Htt and mHtt, respectively, whereas Q23 induced and Q74 induced denote the cells with the proper expression lasted for 48 h.

As shown in Figure 4A, in the case of VDAC preparations isolated from PC12 HD-Q23 cells with or without Htt expression and PC12 HD-Q74 cells without mHtt expression, the obtained distributions of conductance recorded at the membrane potential of +10 mV indicated the dominance of the fully open state with the conductance of about 4 nS in 1M KCl that is consistent with published data. However, in the case of VDAC preparations isolated from PC12 HD-Q74 cells with mHtt expression, the conductance of the open state was decreased that suggested a higher frequency of the channel transition into closed states that might denote the changed voltage-dependence [e.g., Karachitos et al. (34)]. When the voltage-dependence was compared between VDAC preparations isolated from proper control cells and their counterparts with the expression of Htt or mHtt (Figure 4B), no effect was observed for Htt expression, but expression of mHtt affected the parameter distinctly. The effect consisted in the voltage-dependence decrease. Thus, after 48 h of mHtt expression in PC12 HD-Q74 cells, the isolated VDAC preparations with a dominant quantitative content of VDAC1 displayed clear changes of basic VDAC channel characteristic not observed in the case of Htt expression.

## Discussion

Here, we applied the known inducible model of HD comprising cycling PC12 HD-Q23 and PC12 HD-Q74 cells (25) to study the relationship between the cell viability, status of mitochondrial coupling determined in intact cells, and VDAC properties estimated after reconstitution in artificial membranes. Significant difference in the cell viability occurs when Htt and mHtt expression lasted for 48 h (Figure 1). Namely, the viability does not appear to be affected in the case of PC12 HD-Q23 cells but decreases in the case of PC12 HD-Q74 cells. Thus, the duration of mHtt expression allows for observation of its cytotoxic effect.

It has been shown previously for the cell lines that no obvious compromise of the mitochondrial respiratory chain complexes occurs even after 72 h of mHtt expression as determined by their activity measurements in the cell extracts (25). However, it has also been shown that some mitochondria deficits in HD pathomechanism may only be detected in intact cells [e.g., Gouarné et al. (30)]. Our data confirm the assumption as distinct differences in cell respiration and the status of mitochondrial coupling are observed for PC12 HD-Q23 and PC12 HD-Q74 cells when compared to the proper control cells (Figure 2). Interestingly, the differences in basal respiration prelude differences in mitochondrial coupling efficiency (i.e., the state 3 contribution to basal respiration) and mitochondrial coupling capacity (i.e., the FCCP uncoupling capacity). The latter two are observed for Htt and mHtt expression lasted for 48 h whereas the difference in basal respiration occurs already after 24 h of the expression. Thus, manifestation of mHtt cytotoxic effect at the level of cell viability follows in time changes of basal respiration but coincides with changes of the mitochondrial coupling status.

Importantly, the changes of basal respiration determined for PC12 HD-Q23 and PC12 HD-Q74 cells in comparison with proper control cells fluctuate differentially. The fluctuations appear to be supported by the presence of Htt and eliminated by the presence of mHtt. Therefore, it may be suggested that the presence of mHtt may interfere with naturally occurring fluctuations of oxidative metabolism of cycling cells [e.g., Maldonado and Lemasters (14)], which seem to be supported by Htt. It is well known that proper progression of cell cycle requires significant energetic commitments and therefore a timely boost of mitochondrial respiration. Accordingly, it has been shown that the respiratory chain complex I activation and resulting increase of mitochondrial respiration are vital for synchronization between mitochondrial activity and cell-cycle progression (41). Thus, as summarized herein, transient upregulation of mitochondrial respiration is important for successful cell-cycle progression. Correspondingly, according to our preliminary data, when compared with proper control cells the number of PC12 HD-Q74 dividing cells decreases after induction of mHtt expression (not shown). Furthermore, it is proposed that Htt is necessary for mitochondrial structure and function from the earliest stages of embryogenesis (21).

The increased basal respiration observed transiently for PC12 HD-Q23 cells and only initially for PC12 HD-Q74 cells when compared with proper control cells may imply differences in the rate of ATP synthesis and consequently transient differences in ATP content in these cells. The same may apply to the decreased level of basal respiration observed after 48 h of the proper protein synthesis for PC12 HD-Q23 cells but not for PC12 HD-Q74 cells. It might denote that differences in oxidative metabolism between cycling cells expressing Htt and mHtt may oscillate. Accordingly, as summarized by Brustovetsky (42), the available data support two different views on the interaction of mHtt with oxidative metabolism. One group of investigators reports detrimental effects of mHtt on ATP content, whereas another group does not find evidence for such an effect.

The metabolite exchange between mitochondria and cytoplasm is supported by VDAC, which is thus thought to act as a global regulator of the mitochondrial outer membrane permeability and hence of mitochondrial functions [e.g., Homblé et al. (43)]. The protein can be characterized electrophysiologically by determination of the reconstituted channel conductance and voltage-dependence. The calculated distributions of conductances recorded at a membrane potential of +10 mV for VDAC preparations isolated from PC12 HD-Q23 and PC12 HD-Q74 cells (Figure 4) indicate that mHtt expression lasted for 48 h distinctly decreases the conductance of the open state, while expression of Htt does not affect the feature. Thus, the expression of mHtt may promote VDAC closing, a situation known to be accompanied by changes of VDAC selectivity toward cations (39, 40). This, in turn, may influence metabolite exchange between mitochondria and cytoplasm leading to the observed changes of basal respiration and the mitochondrial coupling status.

Importantly, the effect of mHtt on VDAC conductance does not co-occur with the increased voltage-dependence. Conversely, a decrease of the voltage-dependence is observed for the VDAC preparations. Thus, it may be suggested that the decreased conductance of the open state and decreased voltage-dependence reflect VDAC functional changes occurring in cells with expression of mHtt. Correspondingly, we have observed that oxidative stress imposed by depletion of CuZnSOD in *Saccharomyces cerevisiae* cells (Δ*sod1*cells) changes the properties of VDAC (34) in the same direction as that observed for PC12 HD-Q74 cells expressing mHtt. Therefore, it can be speculated that oxidative stress known to be caused by mHtt expression [e.g., Borza (44)] may contribute to the observed changes of VDAC properties. Accordingly, it has been reported that 48 h after induction of mHtt and Htt expression in other PC12 strain lines applied as HD model, PC12 HD-Q103 cells display elevated mitochondrial ROS content when compared with PC12 HD-Q25 or control cells (45). It is, however, still unclear whether the observed changes of the reconstituted VDAC properties may result or/and contribute to oxidative stress.

It has been shown recently that all mammalian VDAC isoforms are able to form channels of comparable characteristic (31, 46, 47). Therefore, it may be speculated that dynamic movement of N-terminal helix defined as the vital element of the voltage-dependence and resulting conductance control [e.g., De Pinto et al. (48)] is impaired in all VDAC isoforms due to the protein modification or interaction with mHtt. However, taking into account the quantitative dominance of VDAC1 in the isolated VDAC preparations (Figure 3), it can be assumed that functional changes of the isoform contribute importantly to the observed effect. Correspondingly, it has been proposed that the mitochondrial outer membrane potential can range between −60 and +60 mV (49) although the issue has not been clarified till now. Nevertheless, the permeability of the membrane can be distinctly changed even in the presence of the small change of the fundamental VDAC feature.

The changes of VDAC properties imposed by the expression of mHtt may be also reflected by changes of a repertoire of interacting proteins. Accordingly, α-tubulin is not detected in VDAC preparations isolated from PC12 HD-Q74 cells with expression of mHtt lasted for 48 h (Table S2 in Supplementary Material). This might denote elimination of its interaction with VDAC that results affects first basal respiration and then the status of mitochondrial coupling and cell viability. Accordingly, free tubulin-dependent differential modulation of mammalian VDAC isoforms is regarded to represent a new mechanism contributing to mitochondrial metabolism and consequently to cell viability (50–53). The modulation is described as a cyclic one and consisting in VDAC reversible blockade. Free tubulin is a heterodimer of α- and β-tubulin, and in cells the dimer is under dynamic equilibrium with microtubules (tubular polymers of tubulin), which is determined by different factors including cell divisions (14, 50). As it is proposed that mHtt enhances oxidative stress and increases protein susceptibility to oxidative modifications (44, 54), and VDAC1 is known to be highly sensitive to oxidative stress [e.g., O’Brien et al. (55); Mello et al. (56); Karachitos et al. (34)], it can be hypothesized that resulting VDAC1 modifications may result in changes of the protein interaction with free tubulin. On the other hand, mHtt has been reported to be associated with microtubules (57). Moreover, free tubulin has been identified as an interacting partner of mHtt and Htt, but the interaction is stronger with mHtt (58). This, in turn, may counteract free tubulin interaction with VDAC1.

Summing up, the presented data indicate that the first measurable mitochondrial symptom of mHtt expression in inducible PC12 cell model of HD is a change of basal respiration and subsequent change of the cell viability. The latter co-occurs with changes of the status of mitochondrial coupling in intact cells and changes of VDAC, being probably mainly VDAC1, properties. Therefore, the data appear to be important for better understanding of cellular processes resulting in cell death. Thus, they may contribute to development of both cytoprotective and cytotoxic strategies of a clinical applicability.

## Author Contributions

AK and HK conceived the study, supervised the performed analyses, and wrote the final version of the manuscript. AK also performed BLM studies. DG supervised PC12 cell culturing, provided samples for BLM and LC–MS/MS, analyzed MS results, and partially drafted the manuscript. KK estimated mitochondria functioning in intact cells. AS performed the viability assays. All the authors read and approved the final version of the manuscript.

## Conflict of Interest Statement

The authors declare that the research was conducted in the absence of any commercial or financial relationships that could be construed as a potential conflict of interest.
