# Supplementary material for: The Association of VDAC with Cell Viability of PC12 Model of Huntington’s Disease
Source: Front Oncol. 2016 Nov 11;6:238. doi: 10.3389/fonc.2016.00238 (PMC5104952; doi:10.3389/fonc.2016.00238)
Supplement: Supplementary file 1 [file Table_1.PDF]

Supplementary Table 1

Cell line	Condition	Basal respiration (nmol O_2/min)	State 3 contribution to basal respiration (%)	FCCP uncoupling capacity (state U/state 4)
PC-12 Q23	4h non-induced	7.15 ± 0.48	44.38 ± 7.13	2.45 ± 0.13
	4h induced	20.14 ± 0.37	55.23 ± 9.69	2.23 ± 0.69
PC-12 Q74	4h non-induced	4.81 ± 1.77	43.48 ± 14.13	3.35 ± 1.08
	4h induced	11.15 0.33	56.21 ± 8.27	2.61 ± 0.95
PC-12 Q23	8h non-induced	5.64 ± 0.49	43.54 ± 9.59	2.31 ± 0.3
	8h induced	4.35 ± 0.05	51.57± 2.02	1.4 ± 0.16
PC-12 Q74	8h non-induced	9.51 ± 1.32	38.59 ± 15.23	3.18 ± 0.61
	8h induced	7.68 ± 0.82	48.52 ± 4.34	2.8 ± 0.69
PC-12 Q23	24h non-induced	2.58 ± 0.21	42.12 ± 7.34	3.02 ± 0.84
	24h induced	7.21 ± 1.07	57.96 ± 9.12	3.45 ± 0.78
PC-12 Q74	24h non-induced	4.09 ± 0.64	49.66 ± 12.43	3.24 ± 0.61
	24h induced	3.79 ± 0.21	68.33 ± 11.23	4.25 ± 0.76
PC-12 Q23	48h non-induced	12.65 ± 0.32	52.5 ± 5.19	6.17 ± 0.23
	48h induced	4.24 ± 0.11	48.66 ± 4.57	4.92 ± 0.27
PC-12 Q74	48h non-induced	7.42 ± 3.07	47.8 ± 9.76	5.27 ± 1.13
	48h induced	8.26 ± 0.96	54.45 ± 8.57	8.23 ± 1.09

Supplementary Table 1. Calculated values of basal respiration, state 3 contribution to basal respiration and FCCP uncoupling capacity for intact PC-12 cells with or without expression of Htt (Q23) and mHtt (Q74).
