# Supplementary material for: The Association of VDAC with Cell Viability of PC12 Model of Huntington’s Disease
Source: Front Oncol. 2016 Nov 11;6:238. doi: 10.3389/fonc.2016.00238 (PMC5104952; doi:10.3389/fonc.2016.00238)
Supplement: Supplementary file 2 [file Table_2.PDF]

Supplementary Table 2

Tubulin type \ Cell line	Q28 non-induced	Q28 induced	Q74 non-induced	Q74 induced
α - tubulin	+	+	+	-
β - tubulin	+	+	+	+

Supplementary Table 2. The presence of α- and β-tubulin monomers in VDAC preparations isolated from PC12 HD-Q23 and PC12 HD-Q73 cells after Htt and mHtt expression, respectively, performed for 48h. Induced, cell with expression of Htt (Q23) or mHtt (Q74); non-induced, cells cultured for 48h but without expression of Htt (Q23) or mHtt (Q74).
