# Supplementary material for: The Association of VDAC with Cell Viability of PC12 Model of Huntington’s Disease
Source: Front Oncol. 2016 Nov 11;6:238. doi: 10.3389/fonc.2016.00238 (PMC5104952; doi:10.3389/fonc.2016.00238)
Supplement: Supplementary file 3 [file Image_1.PDF]

Supplementary Figure 1

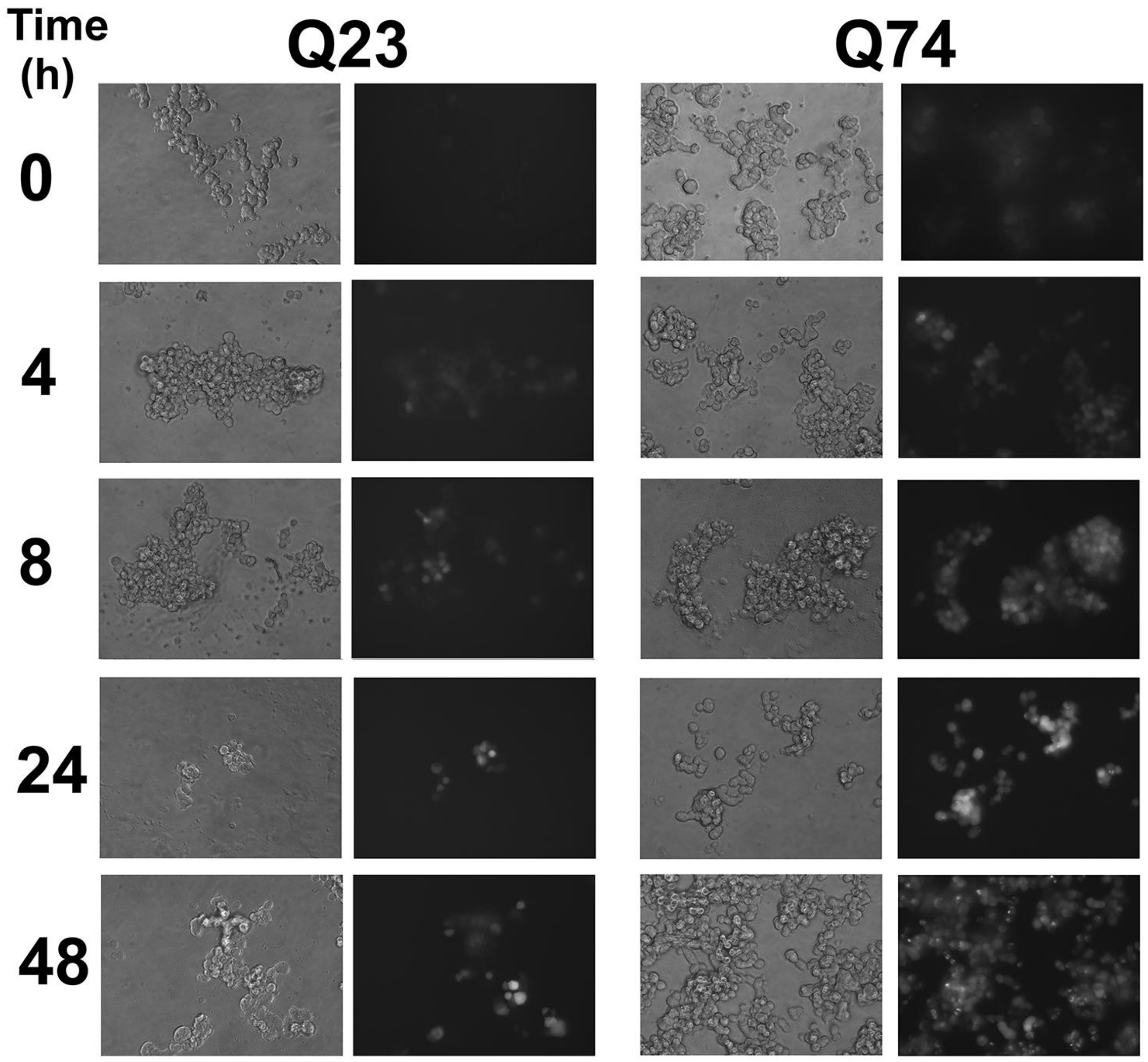

Supplementary Figure 1. Expression of Htt (Q23) and mHtt (Q73) monitored due to their labeling with GFP by ZEISS OBSERVER.Z1 fluorescence microscope.
